# The Texas Health Officers’ Association

**Published:** 1908-07

**Authors:** 


THE
### TEXAS MEDICAL JOURNAL.
Established July, 1885.

F. E. DANIEL, M. D., - - - - Editor, Publisher and Proprietor.

PUBLISHED MONTHLY.—SUBSCRIPTION $1.00 A YEAR.

VOL. XXIV. AUSTIN, JULY, 1908. No. 1.

The publisher is not responsible for the views of contributors.

### Department of Public Health.

### The Texas Health Officers' Association.

Held at Corpus Christi, May 14 and 15, 1908.

### OFFICIAL REPORT OF THE SECRETARY.

(Continued.)

After arrangements for a permanent organization had been completed, President Brumby suggested that all health officers who contemplated leaving Corpus Christi Friday night should read their papers and make their reports forthwith. Accordingly, the following officers either read papers or made reports which will be quoted herein below:

Dr. B. A. Swinney, county health officer, Newton county; Dr. F. A. Miller, of El Paso; Dr. C. W. Truehart, city physician, Galveston; Dr. L. G. Wille, city physician, New Braunfels; Dr. J. H. McCracken, county health officer, Mineral Wells; Dr. J. A. T. Page, Falfurrias; Dr. R. C. Hall, city health officer, Marshall; Dr. E. F. Sharpe, county health officer, Atascosa county; Dr. J. S. Wilkins, of Collingsworth county; Dr. M. J. Phenix, county health officer, Mitchell county; Dr. J. F. Corry, county health officer, Rockwall county; Dr. L. S. Johnson, Cass county; Dr. J. P Mathews, Grayson county; Dr. J. G. Pope, county health officer, Coleman county; Dr. J. J. Adkins, county health officer, Refugio county; Dr. R. W. Skipper, Houston county; Dr. C. H. Reagan, county health officer, Live Oak county; Dr. E. F. Gibson, Walker county; Dr. B. F. Johnson, Wilson county.

*Atascosa County*—The reports made by the different counties in alphabetical order were begun by Dr. E. F. Sharpe, county health officer of Atascosa county. Dr. Sharpe stated that in his county measles, whooping cough, mumps and tuberculosis were prevalent. The county has no detention camp for use in case of pestilential diseases, and in order to declare local quarantine against any premises the commissioners court must act. Dr. Sharpe was impressed with the necessity for providing better accommodations in our county jails for lunatics. Three of these unfortunate persons have killed themselves in the Atascosa county jail. Dr. Sharpe thinks a padded cell would be a valuable adjunct to a jail; he states that his county is largely rural and that, therefore, the physicians are somewhat lax in obeying the vital statistics statute.

*Mitchell County*—After a report by Dr. J. S. Wilkins, of Collingsworth county, Dr. M. J. Phenix, county health officer of Mitchell county, made a report as follows: His county seat is a city of 4000; fortunately, from a sanitary standpoint, Dr. P. C. Coleman is one of the school trustees, and with his support the authorities have succeeded in requiring compulsory vaccination among the school children of Colorado City. It may be due to this fact that Colorado City is free from smallpox, while there are 100 cases in the county surrounding. Dr. Phenix would recommend that all citizens be requird to report contagious diseases, such as diphtheria, measles, scarlatina, whooping cough and smallpox, to the health authorities. In fact, one of the Representatives of Mitchell county in the State Legislature has promised Dr. Phenix to introduce a bill, requiring such reports, in the next State Legislature. Dr. Phenix believes that we must educate the public and in the meantime adopt local regulations to cover sanitary points.

*Rockwall County*—Dr. J. F. Corry, of Rockwall county, has attempted to enforce as far as practicable all the rules and regulations concerning sanitation of public buildings that has been proclaimed by the State Health Officer. He has found teachers to be willing to comply with the law, and the same is true with those in charge of the county jail and courthouse. There are no inspectors of food, meat, milk, etc., although the same are needed. Contagious diseases are reported to the county health officer; since January 1st he has received reports of four cases of smallpox, all of which were in a single focus. The small epidemic was stamped out entirely and every infected house, room or compartment was thoroughly disinfected, as is the invariable rule in his county.

*Cass County*—In the absence of Dr. C. E. Davis, county health officer, Dr. L. S. Johnson, of Atlanta, reports as follows: The county jail and public buildings of Cass county are kept in a sanitary condition. The county health officer co-operates with the State Health Officer actively, and the city council also do all in their power to aid the State authorities in enforcing the sanitary rules. The fact that they are interested is proven by their having paid Dr. Johnson's expenses to this meeting. Vital statistics are reported in full and very thoroughly by the physicians of Cass county, but this can not be said of the midwives. There has been a great deal of tuberculosis in the county, and every doctor looks after his own cases and disinfects after any premises are vacated by a consumptive. There has been a great deal of smallpox of a mild type in Cass; one case occurred in Atlanta and came in contact with many people, but all those who were exposed were vaccinated after exposure, and the disease did not spread at all. Compulsory vaccination has not yet been brought about in Cass county, but the vast majority of the citizens appreciate the great value of vaccination and submit themselves to it voluntarily. There has been a great deal of typhoid in Cass county, a fact possibly due to the lack of waterworks and sewer system; a fact possibly due so far as Atlanta is concerned to the lack of waterworks and sewerage system. Under present conditions scavengers clean up and the individuals pay; the night soil is removed from the city. Later on Dr. Johnson hopes to report Atlanta and Cass county as being in the front rank in sanitary matters.

Grayson county was next heard from, represented by Dr. Matthews, of Howe, in the absence of the county health officer. The people of this county, so the doctor stated, are well posted regarding the importance of sanitation, and are thoroughly in accord with Dr. Brumby in his attempts to protect the public health. Rules as to the sanitation of eating houses and slaughter houses and other places of that character are rigidly enforced. These places are cleaned up each week, and disinfected at intervals. The local authorities did not pay the expenses of their health officer to the Corpus Christi meeting. The record for this county is as follows: Four hundred cases of measles, and not a death; 36 cases of diphtheria, and not a death; 16 cases of tuberculosis, and one death. The local health officer and Dr. Matthews, of Howe, hope to co-operate with Dr. Brumby in the future as in the past.

Coleman county was represented by Dr. J. G. Pope, who stated that this county was also wideawake. They distinfect invariably after premises are vacated by tuberculous individuals. The schools and jail and courthouse are also disinfected, and swept daily after sprinkling with formaldehyde solution, but the commissioners court show a desire to buy a coal tar disinfectant. Slaughter houses in this county are not looked after, and are in an unsanitary condition.

Dr. Brumby here interpolated an explanation of the method adopted in Austin to control slaughter houses. Owing to some uncertainty as to the scope of the word "public building" under the law, there is a little doubt as to just how far the State Health Officer can go in regulating slaughter houses. The matter is now pending in the Attorney General's department. But in Austin the health authorities condemned the slaughter houses, and threatened to obtain an injunction against them as nuisances. If this injunction were obtained, an endeavor would be made to have the meat recently slaughtered and still on hand also condemned. This latter prospect led the butchers to have the slaughter houses cleaned up, in order to avoid the risk of having a lot of meat condemned. Dr. Brumby also answered the question "Who is entitled to membership in the Association?" as follows: All city and county health officers, members of boards of health in incorporated or unincorporated towns in Texas, and other sanitarians who will co-operate with the other members of the Association, and also all State Health Department officials are eligible to registration as members of the Association.

Dr. B. F. Gibson, of Huntsville, next made a short talk. Dr. Gibson is State Prison Physician, and he warned the local health officers about the prevalence of trachoma among the convicts of the State. The local health officer, before shipping a prisoner to the penitentiaries, should examine him for trachoma. If the prisoner has trachoma, he should be segregated and the transfer agent should be notified that it is a trachomatous patient at the time he leaves the place of conviction.

Dr. B. F. Johnson, of Stockdale, Wilson county, was next heard from. He stated that his county had a very efficient county health officer. An epidemic of smallpox had gotten started, and there were about twenty cases, but the prompt action of the school trustees in requiring all the children to be vaccinated saved a more widespread epidemic. In Wilson county all contagious diseases are reported. Vital statistics are reported carefully by the physicians, and the midwives bring the reports to the doctors to get them signed. Mosquitoes are rare in this county.

Judge McNeil Turner here regaled the Association with a stimulating speech on the subject of "State Medicine." The judge said in part: "I am glad to see this rain tonight, because we have had several days of fair weather in Corpus Christi during your convention meeting, and this will show the doctors of Texas that Corpus Christi has all kinds of weather. As for the speeches tonight, I have enjoyed them very much. I am a quarantine crank. I believe in the righteous virtue of the good old shotgun quarantine. And today we look to the members of the Association of Texas Health Officers to protect our State against pestilential invasion. The State Health Department and the local health departments need more and better support from the Legislature. The way to get results is to go after the legislators and candidates. Don't talk to Dr. Brumby about your difficulties. The thing to do is to go home and place the matter up to your candidates for the Legislature."

Judge Turner further said: "One-half of the legislators are cranks, and the other half are fools; you can class me as you please. I have always consistently done all in my power to help the cause of public hygiene and preventive medicine. But one man is powerless. If you examine my record, you will see what I have done. I do not subscribe to the mosquito theory, but I do all I can to help sanitation in every way. The place to get relief is from the State Legislature. There is little use in trying to force the matter through with the aid of the city authorities, because there are too many of them. Oftentimes the city and county officers are desirous of pleasing the people, regardless of whether it is far-sighted policy or not. Hence the way to treat the problem is to go out and elect men that are in line with your ways of thinking. An organized body of men such as the Association of Texas Health Officers can accomplish a great deal. I have thought of a certain plan, which, if constitutional, would give relief. It is to have the State Legislature to create a State Sanitary Commission to have authority in these matters. Then whenever the mayor and aldermen get to disputing the Sanitary Commission could step in and settle the dispute. But is this constitutional? Just now I can not say. At any rate, it would give us uniformity, and that is what we need. At present there is too much diversity of opinion in the different cities. We need uniformity."

The next speaker was Dr. Mayer, Special Inspector of the State Board of Health of Louisiana. After paying his compliments to the Association of Texas Health Officers, Dr. F. J. Mayer went on to state that the present and incoming Governors of Louisiana are both thoroughly in sympathy with the Louisiana system of sanitary education. He reverted to the subject of quarantine and stated that Texas was wise in holding her quarantine privileges to herself, and in not relinquishing them to the Federal authorities.

In support of this view he called attention to the fact that New York has never yet turned over this power to the Federal department. After a most eloquent address, short and impressive, touching on tuberculosis from cow's milk, and typhoid fever, and the mosquito theory of yellow fever transmission, Dr. Mayer concluded his talk.

Adjournment followed until the morning following at 9 o'clock, when the Association met again in the same hall.

Bexar county, represented by Dr. D. Berry, was next heard from as follows:

"Speaking for Bexar county, I take pleasure in reporting great interest in sanitary matters throughout the county generally, and particularly in the city of San Antonio.

"San Antonio is the only incorporated city in the county having an organized sanitary force. It employs a board of health and its clerk, a city health officer and his assistant, a city bacteriologist and sixteen sanitary inspectors, and a fumigator, who does fumigation in the city as ordered by the city health officer. The county employs a fumigator as his services are needed. Of the sixteen inspectors, one inspects fruits and vegetables sold by wholesale, one inspector of restaurant who also inspects the retail fruit and vegetable stands, one detailed in health office, two constantly working the center of the city, and one detailed to collect milk samples and assist the city bacteriologist. Twenty-two scavenger cars, twenty-two push carts, one wagon for hauling dead animals, twelve street sprinklers, and three street sweepers. For the year ending May 31, 1907, $16,399.35 was appropriated for the maintenance of the health department, and $50,640.35 for street cleaning and sanitation in the same length of time. No milk or food inspection is made in the county outside the city confines.

"Since January 1, 1908, the total number of deaths reported in the county was 22; deaths from tuberculosis in the county, 6; meningitis, 1.

"Deaths reported in county clerk's office for city and county: January, 52; February, 103; March, 112; April, 129. During this period eight cases of smallpox were reported in the county, with no deaths; no leprosy, scarlet fever, diphtheria, epidemic cerebrospinal fever, epidemic dysentery or trachoma were reported.

"The quarantine regulations as laid down by the State Health Officer are complied with in every case of smallpox occurring in the county. Regarding diphtheria and scarlet fever, the houses are placarded and disinfected.

"As far as I know the physicians of the city and county are heartily co-operating in the report of contagious diseases. The commissioners court heartily support me in the enforcement of every quarantine regulation and sanitary precaution.

"Compulsory vaccination is not a requirement for entrance to the county public schools, but I made a tour of the county schools some time since and vaccinated every child, whose parents would allow it. Rigid vaccination is enforced in county jail and poorhouse.

"In the county, cases of tuberculosis are not reported, but disinfection is made whenever requested. There is no perceptible increase in tuberculosis among the older residents of the city or county.

"The people in the city and county seem to be alive to the screening of their houses, cisterns, and better drainage.

"I do not favor placing on of quarantine where there are one or two imported cases of yellow fever, provided the disease was recognized early and proper precautions taken, not only with the infected house but of those in the vicinity. I believe that the citizens of San Antonio and Bexar county have confidence in the State Health Officer and local health officers, and will follow any 'safe and sane' method that will insure their protection.

"I have read the proposed sanitary code for unincorporated towns, and believe the citizens of these towns could be interested in the matter when the great good that would accrue from the enforcement of such a code could be shown them.

"The births are not being fully reported in San Antonio and Bexar county, but every month is showing a betterment of conditions. The deaths are fully reported in the city as no burial permit will be issued from the health office without a certificate of death. In the county, I do not believe deaths are fully reported, as physicians report them directly to the county clerk and no burial permit is required. All physicians in the city report deaths to the city health office, and I should say 80 or 90 per cent of the physicians in the county. I believe in San Antonio and Bexar county 75 per cent of physicians report births. My impression is that midwives report births better than physicians."

Comal county, represented by Dr. L. G. Wille, was next heard from as follows:

"Concerning the questions to be answered by the health officers, I will state that our town does well in its sanitary rules. We have only a sanitary committee consisting of four aldermen and the city physician, but we are about to organize a city board of health, and we will adopt the sanitary ordinances as prescribed by the State Health Officer, or those that may be deemed necessary for our city. We have a meat inspector only, who is also the city marshal.

"There has been but one death from typhoid fever in our city during the last three months. This death was due to Christian Science. Regulations on quarantine, isolation and disinfections are strictly enforced.

"The physicians of my town, as well as the city council, cooperate and support the city physician in all his work. We have no sanitary ordinance at present to compel vaccination for school children, but this is to be embodied by the board of health after its organization.

"We have not been in the habit of reporting tuberculosis. We have very few cases of tuberculosis in the city, but now and then a case comes here in our hotels and boarding houses, and some also are taken by private boarding houses.

"Our people are trying, with the aid of the sanitary committee of the board of health, to exterminate mosquitoes.

"I leave it to the county physician to speak about the unincorporated towns and their sanction, although I believe they could be interested as to incorporate them for sanitary purposes under the law.

"*Vital Statistics.*—The county clerk informs me that deaths and births are not fully reported. His estimate of those reported is about 75 per cent. My impression is that the physicians do report, but this low per cent is probably due to the fact that there are born and there die many Mexicans who never have a physician called, and we never hear of the birth or death. Some good neighbors help them with the birth, and the clerk of the county never hears of it. As far as I know, in the city we have but one midwife, and she ought not to be so-called."

Cherokee county, represented by Dr. E. M. Moseley, was next heard from as follows:

"*Sanitation.*—I am glad to say that our people are taking some interest in sanitary affairs. We are making an effort to keep our courthouse and jail in a sanitary condition by constantly cleaning up, and thoroughly disinfecting with such disinfectants as calcium chloride, formaldehyde, hydrargyri bichloride, etc. Slaughter houses and meat markets are not as sanitary as they should be, and are not having the sanitary attention that they should have, but there have been some improvements along this line within the last year. I am sorry to say that there is not the proper attention given to cleaning up day as there should be in the different towns in our county; at the same time can see some improvements all the time, and feel that this will be given more attention in the future than in the past, as our people are becoming more interested on the subject and see the necessity of sanitation more than in the past. Therefore, I am expecting wonderful improvement along this line in the future. There are no towns at all in this county that have a regular sanitary force, only the county health officer and city council see after this business. There are in this county two incorporated towns, namely, Rusk and Jacksonville, and the latter has no city health officer. The mayor attempts to fill the place, and I am acting in the capacity of city health officer of this town. This constitutes the board of health officers for this county.

"*Pure Food.*—There has been no effort in any of the towns in Cherokee county to have a milk and food inspection service.

"*Quarantine.*—I have only been notified of three cases of smallpox in this county, and with one death, although I have heard indirectly that there had been a few others, perhaps a half dozen, but have no assurance of the fact. Have no record of any scarlet fever, leprosy, trachoma, epidemic cerebro-spinal fever. I have records of two cases of diphtheria with one death; one case of chronic dysentery with one death; six cases of typhoid fever with no deaths, and seven cases of tuberculosis with five deaths. The regulations on quarantine isolation and disinfection are not complied with in every instance with scarlet fever and diphtheria; with smallpox it is given more attention, but not as much as should be given. The physicians in my town are co-operating in trying to keep these diseases down, but am sorry to say that there are others in the county who are not co-operating in this work. I am supposed to act only under the instructions of the commissioners court and city council. This is only in cases of smallpox; but have no instructions as to taking charge of cases of diphtheria and scarlet fever. Compulsory vaccination is not required for entrance into the public schools of Cherokee county. The physicians only report cases of tuberculosis in case of deaths, and some not then, and perhaps it is then a month before it is reported, so there are no provisions made for me to follow up and disinfect those houses in case death or removal has taken place. This is only done by the attending physician, and generally in a very careless way, and sometimes not at all. I can not see any perceptible changes in prevalence of tuberculosis in our city or county within the last five years. Some of our people are alive to the necessity of exterminating the mosquito and are screening their houses and cisterns, but this is a very small per cent. I regret to say that a majority of the people give this no thought whatever. However, some of them are gradually awakening to the fact that it is very necessary.

"I am not in favor of placing on a quarantine if I were assured that there were only one or two imported cases of yellow fever in a given town or city in our State, and should be further assured that the disease was recognized early, and all possible precaution taken from the beginning, such as screening and the fumigation of houses infected, and also houses in the same block. I believe that my constituents have sufficient confidence in me, and our State Health Officer to listen to reason, and to stand for a safe and sure method of protection when once convinced of above facts.

"I have read the proposed sanitary code for unincorporated towns, and regret to say that I do not think that the citizens in our unincorporated towns can be interested in co-operating for sanitary purposes under the law governing such. I think the objection would be merely from a financial standpoint.

"*Vital Statistics.*—Approximately the births and deaths reported to our county clerk are about 90 per cent at present. I think about 100 per cent of our doctors are reporting births and deaths, and midwives about 10 or 15 per cent."

